# Associations between fruit and vegetable intake, leisure-time physical activity, sitting time and self-rated health among older adults: cross-sectional data from the WELL study

**DOI:** 10.1186/1471-2458-12-551

**Published:** 2012-07-25

**Authors:** Marita Södergren, Sarah A McNaughton, Jo Salmon, Kylie Ball, David A Crawford

**Affiliations:** 1 Centre for Physical Activity and Nutrition Research, Deakin University, 221 Burwood Hwy, Burwood, VIC, 3125, Australia; 2 Center for Family and Community Medicine, Karolinska Institutet, Huddinge, Sweden

**Keywords:** Lifestyle, Behaviours, IPAQ, Ordinal logistic regression, Interactions

## Abstract

**Background:**

Lifestyle behaviours, such as healthy diet, physical activity and sedentary behaviour, are key elements of healthy ageing and important modifiable risk factors in the prevention of chronic diseases. Little is known about the relationship between these behaviours in older adults. The purpose of this study was to explore the relationship between fruit and vegetable (F&V) intake, leisure-time physical activity (LTPA) and sitting time (ST), and their association with self-rated health in older adults.

**Methods:**

This cross-sectional study comprised 3,644 older adults (48% men) aged 55–65 years, who participated in the Wellbeing, Eating and Exercise for a Long Life (“WELL”) study. Respondents completed a postal survey about their health and their eating and physical activity behaviours in 2010 (38% response rate). Spearman’s coefficient (rho) was used to evaluate the relationship between F&V intake, LTPA and ST. Their individual and shared associations with self-rated health were examined using ordinal logistic regression models, stratified by sex and adjusted for confounders (BMI, smoking, long-term illness and socio-demographic characteristics).

**Results:**

The correlations between F&V intake, LTPA and ST were low. F&V intake and LTPA were positively associated with self-rated health. Each additional serving of F&V or MET-hour of LTPA were associated with approximately 10% higher likelihood of reporting health as good or better among women and men. The association between ST and self-rated health was not significant in the multivariate analysis. A significant interaction was found (ST*F&V intake). The effect of F&V intake on self-rated health increased with increasing ST in women, whereas the effect decreased with increasing ST in men.

**Conclusions:**

This study contributes to the scarce literature related to lifestyle behaviours and their association with health indicators among older adults. The findings suggest that a modest increase in F&V intake, or LTPA could have a marked effect on the health of older adults. Further research is needed to fully understand the correlates and determinants of lifestyle behaviours, particularly sitting time, in this age group.

## Background

It is well established that a healthy diet and physical activity are key factors to prevent chronic diseases and to maintain health throughout the life course [1]. Recent studies have also shown that sedentary behaviour, as distinct from a lack of physical activity, is also an important determinant of health [2-4]. For example, a study from Canada found a dose–response association between sitting time and mortality from all causes and cardiovascular disease, independent of leisure-time physical activity [4]. Although these lifestyle behaviours (healthy diet, physical activity and sedentary behaviour) frequently coexist and are amenable to interventions [5-9], the relationships between them and how they interact with each other have received little attention. In addition, relatively little research has explored these behaviours and their shared association with health indicators in the age group of 55–65 years [6], an increasing group of older adults in transition to retirement [10].

Later adulthood is an important period because the impact of behavioural risk factors increases with age and many chronic diseases such as heart disease, stroke, cancer, and diabetes will present during this life-stage. People reaching retirement will also have access to more leisure-time and greater opportunity to engage in healthy or unhealthy behaviours [11,12]. Existing research shows that many older adults consume too few fruits and vegetables to gain health benefits [13-15]. It is also estimated that older adults are less likely to be sufficiently physically active than younger adults [15,16]. Although younger adults (<40 years) spend more time sitting than do older adults [17], high levels of overall sitting time and TV viewing is associated with greater prevalence of the metabolic syndrome among older adults (>60 years) [18].

Numerous studies have shown that self-rated health is a valid indicator of current health and an independent predictor of later diseases and premature death [19,20]. A single question is often used to measure self-rated health and the response has been shown to be positively correlated with clinical assessments [21,22]. Possible hypotheses for the predictive power of self-rated health include behavioural factors such as physical activity, diet and sedentary behaviours. These are likely to influence metabolism, nutrition and inflammation [21-24], and thereby, behavioural risk factors may precede biological risk factors. Behavioural factors may also be markers of generally risky lifestyles [25] thus, it is important to study modifiable lifestyle behaviours over the life course to prevent chronic disease.

Several studies worldwide have found positive associations between self-rated health and fruit and vegetable intake, physical activity and sedentary behaviour/sitting time among adults [7,9,26-30]. However, most of those studies have examined these lifestyle behaviours separately or combined in an index, overlooking relationships and interactions between them. In addition, previous research has explored health behaviours among adults in general, and there is currently limited research in the field focusing on older adults (age 55–65 years). Therefore, the present study aimed to explore relationships between fruit and vegetable intake, leisure-time physical activity, and sitting time in a large sample of older adults, and to examine their individual and shared association with good self-rated health, controlling for known confounders.

## Methods

### Sample

This study used baseline data provided by older adults aged 55–65 years, who were participants in the Wellbeing, Eating and Exercise for a Long Life (“WELL”) study. The WELL study is a cohort study, which examines the nutrition and physical activity behaviours of Australian older adults. The sample for the WELL study was drawn from the Australian Electoral Commission electoral roll (voting is compulsory in Australia). Between February and April 2010, a sample of adults aged 55–65 years, from urban and rural neighbourhoods in Victoria, Australia were invited to participate in the study and to complete a postal survey covering potential personal, social, and environmental influences relating to eating and physical activity behaviours. All suburbs in urban and rural areas of Victoria were classified according to the Socioeconomic Index for Areas score (SEIFA) which is assigned by the Australian Bureau of Statistics, and divided into tertiles (i.e low, medium and high SEIFA). Fourteen postcodes from each SEIFA tertile (i.e low, medium and high SEIFA) were randomly selected and an equal number of participants from areas within each tertile of SEIFA score were randomly selected. From each suburb, 134 participants (equal numbers of men and women) were selected, resulting in a total sampling pool of 11,256. Of the surveys distributed, 380 were returned as undeliverable and 95 were returned from individuals outside of the 55–65 year age bracket. In total, 4,082 completed surveys were returned (38% response rate). This response rate is similar to what is usually achieved by postal questionnaires of this kind [31]. After exclusion of those who had incomplete or invalid data on the measures included in this study (n = 438) a total of 3,644 (48% men) remained and were included in the analyses. There were statistically significant differences in gender and SEIFA score between non-respondents and respondents (data not shown). Response rates were higher for women than men, and among women, non-respondents were more likely to come from urban areas. Non-respondents were also more likely to live in suburbs with low or medium SEIFA. Among those 3,644 included in the analyses, the percentages for low, medium and high SEIFA were 28.6%, 33.6% and 37.8%, respectively.

### Ethical approval

This research project was approved by the Human Research Ethics Committee of Deakin University (EC-2009-105). All participants gave informed consent in writing.

### Measures

#### Self-rated health and socio-demographic details

Self-rated health was measured by the question: “In general, would you say your health is” [19,20]. The five response alternatives: poor, fair, good, very good and excellent were categorized into three levels: 1) poor/fair, 2) good and 3) very good/excellent. In addition, participants were asked if they had a serious illness, long-term injury or disability that prevented them from being physically active (yes/no). The questionnaire also collected information on gender, weight and height, smoking habits, educational level, marital status, and housing tenure. Body mass index (BMI) was calculated as weight (kg)/height^2^ (m), and classified according to the WHO definition (World Health Organization 2000): normal range as BMI 18.5-24.99, overweight as BMI 25.0–29.9 and obesity as BMI ≥30.0. Those (1.0%) categorized as underweight (BMI <18.5) were included in the normal range category. Marital status was categorized as married/cohabiting, separated/widowed, or single (i.e. never married). Housing tenure was classified into owner, purchaser, or renter/boarder.

#### Fruit and vegetable intake

Fruit and vegetable intakes were assessed by two separate questions: “About how many serves of [fruit/ vegetables] do you usually eat per day”. The eight response options were: I don’t eat fruit/vegetables, less than one serve/day, 1 serve/day, 2 serves/day, 3 serves/day, 4 serves/day, 5 serves/day and 6 or more serves/day. These questions were adapted from the Australian National Nutrition Survey (NNS) [32,33]. They have shown to adequately discriminate between groups with different fruit and vegetable intakes assessed by 24-hour recall, and showed high test-retest reliability (0.85) for both fruit and vegetable intakes [32,33]. Frequency of fruit and vegetable intakes were summed to form a single fruit and vegetable variable (F&V), with total number of servings ranging from 0 (less than 1) to 12 or more servings/day.

#### Leisure-time physical activity

Data on leisure-time physical activity were collected using the self-administered long format of the International Physical Activity Questionnaire (IPAQ-long) [34]. The IPAQ-long asks the respondents to report the frequency and duration (≥10 min) of walking, cycling, and moderate and vigorous intensity physical activity during the past 7 days in four domains: work, active transportation, domestic and garden, and leisure- time. The guidelines obtained from the IPAQ official web page (http://www.ipaq.ki.se) were followed for processing the data. The present analyses used only measures of total time spent in leisure-time walking, moderate- and vigorous-intensity physical activity as some studies have shown that leisure-time physical activity is a better predictor of good self-rated health compared to occupational physical activity [35,36]. Leisure-time physical activity may also provide the best opportunity to intervene compared with occupation and household physical activity. The reported physical activity during leisure-time was converted into metabolic equivalent (MET), and summed into a single continuous LTPA variable (MET-hours/day). The MET weights used in this analysis were 3.3 for walking, 4.0 for moderate intensity physical activity, and 8.0 for vigorous intensity physical activity [37]. Consequently, 1 MET-hour corresponds to about 18 minutes of walking, 15 minutes of moderate (e.g. brisk walking), or 7.5 minutes of vigorous LTPA (e.g. running at 8 km/hrs) [37].

#### Sitting time

Data on sitting time were also collected from the IPAQ-long [17]. Respondents are asked to report time spent sitting while at work, at home, while doing study, and in leisure-time during the last 7 days. Total time spent sitting on weekdays and weekend days were summed and presented as average daily time spent sitting (ST) (hours/day).

### Statistical analysis

Tests of differences between women and men, in the distribution of the descriptive variables (categorical) were performed using a Chi-square-test, and for age (continuous) by one-way analysis-of-variances (ANOVA). ANOVA was also used to examine differences between women and men in reported F&V intake and ST, whereas the Kruskal-Wallis test was used for the LTPA variable (due to skewed nature of the data). The relations between F&V, LTPA and ST were tested using Spearman’s rank correlation coefficient. Ordinal logistic regression models, with self-rated health (poor/fair, good, very good/excellent) as the outcome, were used to investigate the association with F&V, LTPA and ST. Two models were calculated (bivariate and multivariate), stratified by sex. The results are presented as odds ratios (ORs) with 95% confidence interval (CI).

Previous studies have recognized age, gender, BMI, smoking, marital status and education or other markers of socioeconomic position, such as housing tenure as correlates of self-rated health [28-30]. Several of these factors have also been shown to be correlated with healthy behaviours [25,38-40]. In the present study, only those variables found to have a significant bivariate association with self-rated health were included in the multivariate model as possible confounders (BMI, smoking habits, marital status, education, housing tenure, long-term illness). Probably due to the narrow age range included, age was neither significantly associated with self-rated health in the bivariate analysis (p = 0.81, p = 0.27 for women and men respectively) nor did its inclusion change the parameters for the variables of interest or improve the model fit. The models were therefore not adjusted for age. In addition, the two variables income and employment were also tested as possible confounders but were omitted from the analyses as they were not significantly associated with the outcome variables. When assessing the association between F&V, LTPA, ST and self-rated health, effect modifications were evaluated in the regression model by testing interaction terms between F&V*LTPA, F&V*ST and LTPA*ST. Statistical analysis was conducted using Stata version 11.0 (Stata Corporation, College Station TX, USA). A p-value of less than 0.05 was considered statistically significant.

## Results

A greater proportion of women rated their health as very good/excellent compared to men (Table 1). The results also showed that 57% of the women and 72% of the men were overweight or obese. Given the significant differences in self-rated health and other factors between women and men, the regression analyses were stratified by sex. Women on average reported consuming one more serving of F&V per day and approximately 30 minutes less time spent sitting per day than did men (Table 2). The small, but significant differences in median LTPA (0.3 MET-hours/day) between men and women corresponds to approximately 38 minutes of walking per week. There were weak but significant correlations between F&V intake and LTPA, and between LTPA and ST, in both women and men (Table 3). A significant inverse correlation between F&V intake and ST was only found for women.

**Table 1:** Mean and distribution of the individual variables (percentages)

**Variables**	**Total**	**Women**	**Men**	**P-value**^**a**^
***Sample size (n)***	3,644	1,892	1,752	
*Mean age*	60.2	60.2	60.2	0.865
*(SD)*	(3.2)	(3.2)	(3.1)	
***Self-rated health (%)***				0.004
Very good/excellent	48.4	51.0	45.8	
Good	39.9	38.5	41.3	
Fair/poor	11.7	10.5	12.9	
***Body mass index (BMI) (%)***				< 0.001
< 25 kg/m^2^	36.2	43.5	28.3	
25–29.9 kg/m^2^	39.8	32.8	47.5	
≥ 30 kg/m^2^	24.0	23.7	24.3	
***Smoking habits (%)***				< 0.001
Never smoker	50.2	56.4	43.5	
Former smoker	37.8	32.9	43.2	
Daily smoker	12.0	10.7	13.3	
***Education (%)***				< 0.001
Up to 10 years	36.1	40.3	31.7	
12 years/trade/certificate	35.9	32.4	39.7	
University degree	27.9	27.3	28.6	
***Marital status (%)***				< 0.001
Married/cohabiting	78.8	75.2	82.7	
Separated/widowed	16.1	20.5	11.3	
Single	5.1	4.3	6.0	
***Housing tenure (%)***				0.448
Owner	69.0	69.9	68.1	
Purchaser	20.6	19.8	21.4	
Renter/boarder	10.4	10.3	10.5	
***Long- term illness (%)***				
No	78.8	79.8	77.9	0.160
Yes	21.2	20.2	22.1	

Note ^a^ Statistical differences by sex: One-way ANOVA and Chi-square test.

**Table 2:** Reported F&V intake, LTPA and ST presented as daily mean and median, respectively (n = 3,644)

	**Total**	**Women**	**Men**	**p-value**^**a**^
**F&V** (servings/day)				< 0.001
Mean ± SD	4.4 ± 2.2	5.0 ± 2.1	3.9 ± 2.2	
Median (CI)	4.0 (4.0-4.0)	5.0 (5.0-5.0)	4.0 (4.0-4.0)	
**LTPA** (MET-hours/day)				0.005
Mean ± SD	2.5 ± 3.4	2.5 ± 3.3	2.5 ± 3.6	
Median (CI)	1.4 (1.2-1.4)	1.4 (1.4-1.6)	1.1 (1.0-1.4)	
**ST** (hours/day)				0.005
Mean ± SD	5.8 ± 2.9	5.6 ± 2.8	6.1 ± 3.0	
Median (CI)	5.1 (5.0-5.3)	5.0 (5.0-5.1)	5.4 (5.3-5.6)	

SD - Standard deviation. CI - 95% Confidence Interval.

Note ^a^ Statistical differences by sex: One-way ANOVA and Kruskal-Wallis test.

**Table 3:** Spearman’s rank order correlations between F&V intake, LTPA and ST (n = 3,644)

	**F&V – LTPA (p-value)**	**F&V – ST (p-value)**	**LTPA –ST (p-value)**
**Women**	0.196 (< 0.001)	−0.066 (0.004)	−0.110 (< 0.001)
**Men**	0.143 (< 0.001)	−0.022 (0.360)	−0.065 (0.006)

Table 4 shows the ordinal logistic regression models predicting the odds of reporting health as good or better (very good/excellent), stratified by sex. F&V intake and LTPA were positively associated with self-rated health, whereas ST was negatively associated with self-rated health. In the multivariate model, each additional daily serving of F&V was associated with higher odds of reporting health as good or better in both women and men, by 9% and 10% respectively. The odds of reporting health as good or better were also higher for each additional daily MET-hour (e.g. 15 min brisk walk) of LTPA in both women and men. The negative (bivariate) association between ST and self-rated health were no longer significant in the multivariate analyses.

**Table 4:** Odds ratios (ORs) with 95% confidence intervals (CIs) for reporting self-rated health as good or better (n = 3,644)

		**Bivariate model**		**Multivariate model**^**a**^	
		**OR(CI)**		**OR(CI)**	
***Variable***	**Level**	**Women**	**Men**	**Women**	**Men**
***F&V (servings/day)***		1.15 (1.11-1.20)	1.16 (1.11-1.21)	1.09 (1.04-1.14)	1.10 (1.05-1.16)
***LTPA (MET-hours/day)***		1.15 (1.11-1.20)	1.16 (1.12-1.21)	1.08 (1.04-1.12)	1.10 (1.06-1.14)
***ST (hours/day)***		0.95 (0.92-0.98)	0.94 (0.91-0.97)	0.98 (0.95-1.02)	0.96 (0.93-1.00)
***Body Mass Index***	< 25 kg/m^2^	1	1	1	1
*(BMI)*	25–29.9 kg/m^2^	0.63 (0.52-0.77)	0.77 (0.62-0.96)	0.70 (0.56-0.86)	0.84 (0.66-1.06)
	≥ 30 kg/m^2^	0.35 (0.28-0.44)	0.28 (0.21-0.36)	0.54 (0.42-0.70)	0.36 (0.27-0.47)
***Smoking habits***	Never smoker	1	1	1	1
	Former smoker	0.86 (0.71-1.04)	0.68 (0.56-0.83)	0.99 (0.81-1.22)	0.79 (0.64-0.98)
	Daily smoker	0.44 (0.33-0.59)	0.33 (0.25-0.43)	0.65 (0.47-0.89)	0.48 (0.35-0.65)
***Education***	Up to 10 years	1	1	1	1
	12 years/trade/certificate	1.14 (0.93-1.39)	1.37 (1.11-1.69)	1.01 (0.82-1.25)	1.12 (0.89-1.41)
	University	1.98 (1.58-2.48)	2.38 (1.88-3.01)	1.42 (1.11-1.82)	1.54 (1.19-2.00)
***Marital status***	Married/cohabiting	1	1	1	1
	Separated/widowed	0.74 (0.59-0.93)	0.69 (0.52-0.91)	0.93 (0.73-1.18)	0.78 (0.57-1.08)
	Single	0.54 (0.35-0.84)	0.59 (0.41-0.86)	0.63 (0.38-1.05)	0.55 (0.37-0.82)
***Housing tenure***	Renter/boarder	1	1	1	1
	Purchaser	2.14 (1.51-3.03)	1.79 (1.28-2.51)	1.73 (1.19-2.52)	1.11 (0.76-1.62)
	Owner	2.30 (1.69-3.12)	1.94 (1.44-2.61)	1.59 (1.14-2.23)	1.14 (0.81-1.58)
***Long-term illness***	No	1	1	1	1
	Yes	0.12 (0.09-0.16)	0.13 (0.10-0.17)	0.16 (0.12-0.21)	0.17 (0.13-0.23)

Note ^a^ Multivariate model – adjusted for all the independent variables (including F&V, LTPA and ST) simultaneously.

**Figure 1:**
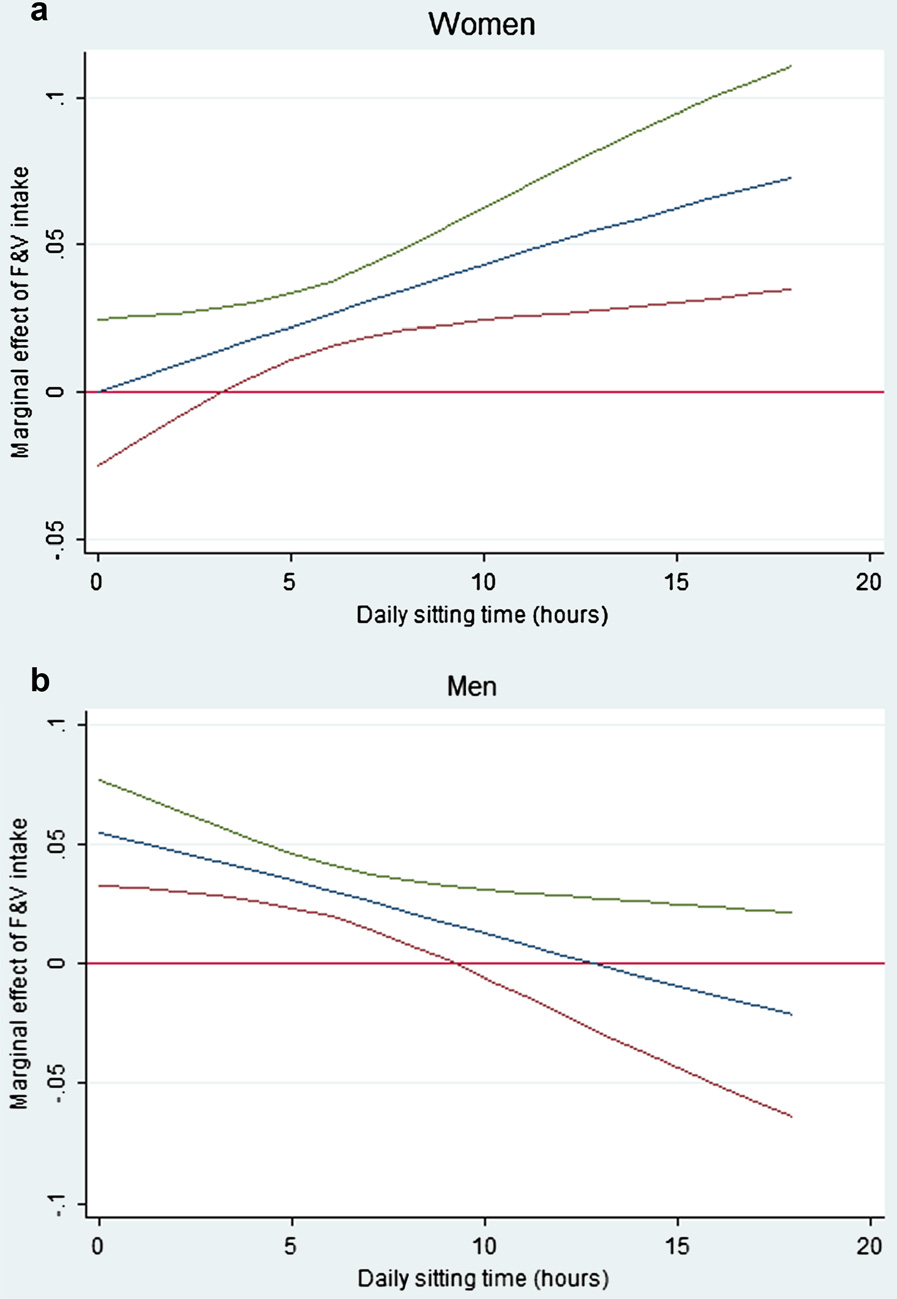
**Interaction effect**^**a**^**with LTPA held at mean, with 95% confidence intervals (CIs), women.** Note ^a^ Change in probability of rating health as good or better for one unit change in F&V intake at different values of ST.

On testing interaction terms in the multivariate models it was found that ST acted as a moderator of the association between F&V intake and self-rated health, in opposite directions, for women and men (OR = 1.02; 95% CI:1.00-1.03 and OR = 0.98; 95% CI: 0.96-0.99, respectively). This indicates that increased ST strengthens the association between F&V and self-rated health in women, and weakens it in men. Figures 1a and1b shows that this effect was statistically significant above 5 hours of ST in women, and below 10 hours of ST in men. Plotting the estimates of the marginal effects of F&V, along with 95% confidence intervals and LTPA held at mean, showed similar result for both cut-points of self-rated health (fair/poor vs. good or better, good vs. better). Therefore, only one cut-point (good vs. better) is shown in Figures 1a and 1b. Modification effects by F&V and LTPA were also tested, but the results were not statistically significant.

## Discussion

The findings show that each additional serving of fruit and vegetable or 15 min brisk walk per day, was associated with approximately 10% increase in the odds of reporting health as good or better among women and men. The effect size remained statistically significant when all three lifestyle behaviours were included in the model, and after adjusting for BMI, smoking, long-term illness and socio-demographic characteristics (education, marital status and housing tenure). Taking into consideration the burden of chronic diseases, an increase in F&V intake and LTPA of this size may have a substantial impact on public health. However, these cross-sectional findings need to be confirmed longitudinally.

Recent large prospective studies show that compliance with multiple healthy behaviours is associated with the reduced risk of mortality in a dose–response way [5,6]. For example, the mortality risk associated with compliance with four compared to zero health behaviours (never smoked, healthy diet, adequate physical activity and moderate alcohol use) was equivalent to being up to 14 years younger in chronological age [5,6]. The study by Ford et al. also showed that the combined impact of a healthy diet and adequate physical activity reduced the risk of all-cause mortality by 25% [5]. Another longitudinal study, examining health behaviours and quality of life, suggest that physical activity is the key factor, superior to fruit and vegetable consumption and sedentary time (viewing), when influencing individual’s mental health and quality of life [41]. On the other hand, an untargeted increase in physical activity has been reported in interventions to promote fruit and vegetable consumption [42]. Furthermore, randomized control trials have shown that individuals that adopted one healthy behaviour are more likely to adopt another healthy behaviour, and that there are even greater benefits with reducing bundle risk behaviours simultaneously [43,44]. Although these studies were among adults in general and did not focus specifically on older adults aged 55–65 years, the results give justification for looking at health behaviours in combination, and the importance to examine both additive and synergistic effects on health outcomes. They also give insights into the quantity and quality of life that can be potentially gained when adopting healthy lifestyles. The results from the present study show similar association for F&V and LTPA with self-rated health, and provide further support that even small differences in lifestyle behaviours may make a big difference to health in the population.

Worldwide it is well-recognised that the risks for chronic disease are escalating, and the future burden (social and economic) will be largely determined by current lifestyle behaviours [10]. In Australia, a majority of older adults aged 55–64 years have three or more behavioural risk factors for chronic disease, and low intake of fruit and vegetables together with lack of physical activity is the most common combination [45]. Hence, to bring about an increase in F&V intake or/and LTPA could result in postponement of age-associated diseases which allows independent living for a longer period of time [1,10]. However, for optimal promotion of healthy lifestyles in older adults, we need to understand both prevalence of health behaviours and associations among such behaviours. The low correlation coefficients, between F&V intake, LTPA and ST in the present study, indicate that they have little if any linear correlation, or that confounding variables might be involved. A range of personal, social, and environmental factors, (above and beyond the socio-demographic factors included in the present study), are important influences on food consumption, physical activity and sedentary behaviours [17,46]. A better understanding of these influences among older adults is necessary to take into account the specific life-stage context.

Considering the growing evidence of an association between sedentary behaviour and different health outcomes [2,4], the lack of an association between ST and self-rated health was unexpected. One reason could be that ST in older adults might be more related with objective health indicators [4]. Another explanation could be the measure of ST. Although the sitting measure in the IPAQ has acceptable reliability and validity among adults aged 18–65 years [17], perhaps the measure did not perform so well among Australian adults in this age group. A limitation of the sitting measure in IPAQ is that it does not distinguish between sitting in different domains, such as work, transportation, domestic and garden, and leisure- time. Therefore, associations between discretionary sitting and self-rated health could not be examined, as was done for LTPA. When specific sedentary behaviours are examined the most common sedentary leisure-time behaviour is TV viewing time [8].

The positive and negative interaction effect (for women and men, respectively) between ST and F&V was also surprising. This means, that the simultaneous influence of ST and F&V on self-rated health is not additive. Instead, the association between F&V and self-rated health is dependent on ST and additional time spent sitting affects this association differently in women and men. Therefore, among men the association between F&V and self-rated health strengthens with less time spent sitting (<10 hr/day) (Figure 1b). However, the reverse effect for added time spent sitting (>5 hr/day) in women are more difficult to explain (Figure 1a). It is possible that the different associations for men and women are a result of different patterns of sitting time, for example, prolonged periods versus intermittent sitting bouts however there is limited research on sitting time among this age group [3,8]. Objective measures of sedentary time in US shows that male older adults (>60 years) are more sedentary than their female counterparts [47]. However, an international comparative study of sitting time among adults in 20 countries did not find any gender differences [17]. Consequently, more research is needed to clarify associations with sedentary behaviour in this age group.

One limitation of this study is the use of self-reported data which may be hampered by recall biases such as social desirability (including cultural and gender differences), and over- or under-reporting [48-50]. The modest response rate, which can introduce bias into study results, is also acknowledged. Although there were some differences between respondents and non-respondents, the distribution did not differ compared to Australian national data for self-rated health and health risk factors (smoking, BMI, fruit and vegetable consumption, exercise level) in the same age group [15]. Even if selection bias cannot be ruled out (e.g. those that agreed to participate could have healthier behaviours than nonparticipants), the data were obtained from a relatively large, random sample of older adults sampled from the Australian Electoral Commission electoral roll (voting is compulsory for person aged 18 years and over), which limits the risk of self-selection bias. The cross-sectional design and inability to determine causality of effect is also a limitation. Thus, it is difficult to assess which factors are determinants and which are consequences in the association between F&V intake, LTPA, ST and self-rated health. Though we found no association between long-term illness and physical activity, it cannot be excluded that some individuals do not take part in physical activity and/or might be more sedentary due to various health problems, including mental health problems. However, the WELL study is designed as a prospective cohort so we will be able to investigate the findings longitudinally. Furthermore, the ability to concurrently explore a set of key factors that may have an impact on older adult’s future health, with sufficient power and adjusted for important confounders is a strength of this study. In light of an acceleration of the ageing of the global population [10], and bearing in mind that self-rated health is strongly associated with successful healthy ageing, identifying its determinants is of importance for understanding the underpinnings of good health in later life.

## Conclusions

A better understanding of the relationship between lifestyle behaviours can accelerate our efforts to improve health outcomes for which diet, physical activity and sedentary behaviours are risk factors. The present study contributes to the scarce literature related to lifestyle behaviours and their association with health indicators among older adults. The findings suggest that a modest increase in F&V intake, or LTPA could have a marked effect on the health of older adults. Further research is needed to fully understand the correlates and determinants of lifestyle behaviours, particular sitting time, in this age group due to the lack of current research and potential health benefits from lifestyle changes.

## Competing interests

The authors declare that they have no competing interests.

## Authors’ contributions

MS was the primary author and conducted the analyses in consultation with all authors. All authors helped to conceptualize ideas, interpret findings, and review drafts of the manuscript. SM, JS, KB and DC designed the study and contributed to the development and implementation of the study. All authors gave final approval of the version to be published.

## Pre-publication history

The pre-publication history for this paper can be accessed here:

http://www.biomedcentral.com/1471-2458/12/551/prepub

## References

[B1] WHO:2008–2013 Action plan for the global strategy for the prevention and control of noncommunicable diseases 2008 Geneva, Switzerland: World Health Organisation

[B2] Hamilton M Healy G Dunstan D Zderic T Owen N Too little exercise and too much sitting: Inactivity physiology and the need for new recommendations on sedentary behavior Current Cardiovascular Risk Reports 2008 2 4 292 298 10.1007/s12170-008-0054-8 22905272 PMC3419586

[B3] Tremblay MS Colley RC Saunders TJ Healy GN Owen N Physiological and health implications of a sedentary lifestyle Appl Physiol Nutr Metab 2010 35 6 725 740 10.1139/H10-079 21164543

[B4] Katzmarzyk PT Church TS Craig CL Bouchard C Sitting time and mortality from all causes, cardiovascular disease, and cancer Med Sci Sports Exerc 2009 41 5 998 1005 10.1249/MSS.0b013e3181930355 19346988

[B5] Ford ES Zhao G Tsai J Li C Low-Risk Lifestyle Behaviors and All-Cause Mortality: Findings From the National Health and Nutrition Examination Survey III Mortality Study Am J Public Health 2011 101 10 1922 1929 10.2105/AJPH.2011.300167 21852630 PMC3222361

[B6] Khaw K-T Wareham N Bingham S Welch A Luben R Day N Combined Impact of Health Behaviours and Mortality in Men and Women: The EPIC-Norfolk Prospective Population Study PLoS Medicine 2008 5 1 e12 10.1371/journal.pmed.0050012 18184033 PMC2174962

[B7] Pisinger C Toft U Aadahl M Glumer C Jorgensen T The relationship between lifestyle and self-reported health in a general population: the Inter99 study Prev Med 2009 49 5 418 423 10.1016/j.ypmed.2009.08.011 19716843

[B8] Sugiyama T Healy GN Dunstan DW Salmon J Owen N Joint associations of multiple leisure-time sedentary behaviours and physical activity with obesity in Australian adults Int J Behav Nutr Phys Act 2008 5 35 10.1186/1479-5868-5-35 18590570 PMC2459202

[B9] Tsai J Ford ES Li C Zhao G Pearson WS Balluz LS Multiple healthy behaviors and optimal self-rated health: findings from the 2007 Behavioral Risk Factor Surveillance System Survey Prev Med 2010 51 3–4 268 274 20647019 10.1016/j.ypmed.2010.07.010

[B10] World Population Ageing 2007 2007 New York, N.Y

[B11] Evenson KR Rosamond WD Cai J Diez-Roux AV Brancati FL Influence of retirement on leisure-time physical activity: the atherosclerosis risk in communities study Am J Epidemiol 2002 155 8 692 699 10.1093/aje/155.8.692 11943686

[B12] Nooyens AC Visscher TL Schuit AJ van Rossum CT Verschuren WM van Mechelen W Seidell JC Effects of retirement on lifestyle in relation to changes in weight and waist circumference in Dutch men: a prospective study Public Health Nutr 2005 8 8 1266 1274 16372922 10.1079/phn2005756

[B13] Tamers SL Agurs-Collins T Dodd KW Nebeling L US and France adult fruit and vegetable consumption patterns: an international comparison Eur J Clin Nutr 2009 63 1 11 17 10.1038/ejcn.2008.2 18270525

[B14] Dehghan M Akhtar-Danesh N 2011 Merchant AT: Factors associated with fruit and vegetable consumption among adults. J Hum Nutr Diet 10.1111/j.1365-277X.2010.01142.x 21332835

[B15] National Health Survey - Summary of results 2009 Canberra, ACT: ABS

[B16] Mealing NM Bowles HR Merom D Bauman A Impact of scoring algorithm on physical activity prevalence estimates in Australian adults J Sci Med Sport 2011 14 1 27 32 10.1016/j.jsams.2010.05.003 20594908

[B17] Bauman A Ainsworth BE Sallis JF Hagstromer M Craig CL Bull FC Pratt M Venugopal K Chau J Sjostrom M The Descriptive Epidemiology of Sitting A 20-Country Comparison Using the International Physical Activity Questionnaire (IPAQ) Am J Prev Med 2011 41 2 228 235 10.1016/j.amepre.2011.05.003 21767731

[B18] Gardiner PA Healy GN Eakin EG Clark BK Dunstan DW Shaw JE Zimmet PZ Owen N Associations between television viewing time and overall sitting time with the metabolic syndrome in older men and women: the Australian Diabetes, Obesity and Lifestyle study J Am Geriatr Soc 2011 59 5 788 796 10.1111/j.1532-5415.2011.03390.x 21568949

[B19] DeSalvo KB Bloser N Reynolds K He J Muntner P Mortality prediction with a single general self-rated health question. A meta-analysis J Gen Intern Med 2006 21 3 267 275 10.1111/j.1525-1497.2005.00291.x 16336622 PMC1828094

[B20] Idler EL Benyamini Y Self-rated health and mortality: a review of twenty-seven community studies J Health Soc Behav 1997 38 1 21 37 10.2307/2955359 9097506

[B21] Lekander M Elofsson S Neve IM Hansson LO Unden AL Self-rated health is related to levels of circulating cytokines Psychosom Med 2004 66 4 559 563 10.1097/01.psy.0000130491.95823.94 15272103

[B22] Nixon Andreasson A Jernelov S Szulkin R Unden AL Brismar K Lekander M Associations between leptin and self-rated health in men and women Gend Med 2010 7 3 261 269 10.1016/j.genm.2010.05.001 20638631

[B23] Oliveira A Rodriguez-Artalejo F Lopes C The association of fruits, vegetables, antioxidant vitamins and fibre intake with high-sensitivity C-reactive protein: sex and body mass index interactions Eur J Clin Nutr 2009 63 11 1345 1352 10.1038/ejcn.2009.61 19623199

[B24] Healy GN Dunstan DW Salmon J Cerin E Shaw JE Zimmet PZ Owen N Breaks in sedentary time: beneficial associations with metabolic risk Diabetes Care 2008 31 4 661 666 10.2337/dc07-2046 18252901

[B25] Laaksonen M Talala K Martelin T Rahkonen O Roos E Helakorpi S Laatikainen T Prattala R Health behaviours as explanations for educational level differences in cardiovascular and all-cause mortality: a follow-up of 60 000 men and women over 23 years Eur J Public Health 2008 18 1 38 43 10.1093/eurpub/ckm051 17569702

[B26] Han MA Kim KS Park J Kang MG Ryu SY Association between levels of physical activity and poor self-rated health in Korean adults: The Third Korea National Health and Nutrition Examination Survey (KNHANES), 2005 Public Health 2009 123 10 665 669 10.1016/j.puhe.2009.08.005 19854457

[B27] Wendel-Vos GC Schuit AJ Tijhuis MA Kromhout D Leisure time physical activity and health-related quality of life: cross-sectional and longitudinal associations Qual Life Res 2004 13 3 667 677 15130029 10.1023/B:QURE.0000021313.51397.33

[B28] Haseli-Mashhadi N Pan A Ye X Wang J Qi Q Liu Y Li H Yu Z Lin X Franco OH Self-Rated Health in middle-aged and elderly Chinese: distribution, determinants and associations with cardio-metabolic risk factors BMC Public Health 2009 9 368 10.1186/1471-2458-9-368 19788754 PMC2760533

[B29] Molarius A Berglund K Eriksson C Lambe M Nordstrom E Eriksson HG Feldman I Socioeconomic conditions, lifestyle factors, and self-rated health among men and women in Sweden Eur J Public Health 2007 17 2 125 133 10.1093/eurpub/ckl070 16751631

[B30] Barros MB Zanchetta LM Moura EC Malta DC Self-rated health and associated factors, Brazil, 2006 Rev Saude Publica 2009 43 Suppl 2 27 37 19936496 10.1590/s0034-89102009000900005

[B31] Edwards P Roberts I Clarke M DiGuiseppi C Pratap S Wentz R Kwan I Cooper R Methods to increase response rates to postal questionnaires Cochrane Database Syst Rev 2007 2 MR000008 17443629 10.1002/14651858.MR000008.pub3

[B32] McLennan W Podger A National Nutrition Survey Users' Guide Catalogue No 48010 1998 Canberra, ACT: Australian Bureau of Statistics

[B33] Rutishauser IHE Webb K Abraham B Allsopp R Evaluation of short dietary questions from the 1995 National Nutrition Survey. National Food and Nutrition Monitoring and Surveillance Project 2001 Canberra, ACT: Commonwealth Department of Health and Aged Care

[B34] Craig CL Marshall AL Sjostrom M Bauman AE Booth ML Ainsworth BE Pratt M Ekelund U Yngve A Sallis JF International physical activity questionnaire: 12-country reliability and validity Med Sci Sports Exerc 2003 35 8 1381 1395 10.1249/01.MSS.0000078924.61453.FB 12900694

[B35] Kaleta D Makowiec-Dabrowska T Dziankowska-Zaborszczyk E Jegier A Physical activity and self-perceived health status Int J Occup Med Environ Health 2006 19 1 61 69 10.2478/v10001-006-0005-x 16881600

[B36] Okano G Miyake H Mori M Leisure time physical activity as a determinant of self-perceived health and fitness in middle-aged male employees J Occup Health 2003 45 5 286 292 10.1539/joh.45.286 14646269

[B37] Ainsworth BE Haskell WL Whitt MC Irwin ML Swartz AM Strath SJ O'Brien WL Bassett DR Schmitz KH Emplaincourt PO Compendium of physical activities: an update of activity codes and MET intensities Med Sci Sports Exerc 2000 32 9 Suppl S498 S504 10993420 10.1097/00005768-200009001-00009

[B38] Margetts BM Rogers E Widhal K de Winter AM R Zunft HJ Relationship between attitudes to health, body weight and physical activity and level of physical activity in a nationally representative sample in the European Union Public Health Nutr 1999 2 1A 97 103 10933629 10.1017/s1368980099000142

[B39] Sodergren M Sundquist J Johansson SE Sundquist K Physical activity, exercise and self-rated health: a population-based study from Sweden BMC Public Health 2008 8 352 10.1186/1471-2458-8-352 18840294 PMC2576235

[B40] Ball K Crawford D Socio-economic factors in obesity: a case of slim chance in a fat world? Asia Pac J Clin Nutr 2006 15 Suppl 15 20 16928657

[B41] Chai W Nigg CR Pagano IS Motl RW Horwath C Dishman RK Associations of quality of life with physical activity, fruit and vegetable consumption, and physical inactivity in a free living, multiethnic population in Hawaii: a longitudinal study Int J Behav Nutr Phys Act 2010 7 83 10.1186/1479-5868-7-83 21092223 PMC2996342

[B42] Berrigan D Dodd K Troiano RP Krebs-Smith SM Barbash RB Patterns of health behavior in U.S. adults Prev Med 2003 36 5 615 623 10.1016/S0091-7435(02)00067-1 12689807

[B43] Johnson SS Paiva AL Cummins CO Johnson JL Dyment SJ Wright JA Prochaska JO Prochaska JM Sherman K Transtheoretical model-based multiple behavior intervention for weight management: effectiveness on a population basis Prev Med 2008 46 3 238 246 10.1016/j.ypmed.2007.09.010 18055007 PMC2327253

[B44] Spring B Schneider K McFadden H Vaughn J Kozak AT Smith M Moller AC Epstein L Russell SW DeMott A Make Better Choices (MBC): Study design of a randomized controlled trial testing optimal technology-supported change in multiple diet and physical activity risk behaviors BMC Public Health 2010 10 586 10.1186/1471-2458-10-586 20920275 PMC2955698

[B45] AIHW:Risk factors contributing to chronic disease 2012 Canberra: AIHW: Australian Institute of Health and Welfare

[B46] Ball K People, places…and other people? Integrating understanding of intrapersonal, social and environmental determinants of physical activity J Sci Med Sport 2006 9 5 367 370 10.1016/j.jsams.2006.06.010 16857424

[B47] Matthews CE Chen KY Freedson PS Buchowski MS Beech BM Pate RR Troiano RP Amount of time spent in sedentary behaviors in the United States, 2003–2004 Am J Epidemiol 2008 167 7 875 881 10.1093/aje/kwm390 18303006 PMC3527832

[B48] Ferrari P Friedenreich C Matthews CE The role of measurement error in estimating levels of physical activity Am J Epidemiol 2007 166 7 832 840 10.1093/aje/kwm148 17670910

[B49] Richardson MT Ainsworth BE Jacobs DR Leon AS Validation of the Stanford 7-day recall to assess habitual physical activity Ann Epidemiol 2001 11 2 145 153 10.1016/S1047-2797(00)00190-3 11164131

[B50] Rzewnicki R Vanden Auweele Y De Bourdeaudhuij I Addressing overreporting on the International Physical Activity Questionnaire (IPAQ) telephone survey with a population sample Public Health Nutr 2003 6 3 299 305 12740079 10.1079/PHN2002427

